# INSIGHTS ON THE CLINICAL NURSE ROLE IN IMPLEMENTING WHAT MATTERS FOR OLDER ADULTS IN ACUTE CARE

**DOI:** 10.1093/geroni/igad104.0191

**Published:** 2023-12-21

**Authors:** Murad Taani, Mary Hook

**Affiliations:** University of Wisconsin-Milwaukee, Milwaukee, Wisconsin, United States; Advocate Health, Milwaukee, Wisconsin, United States

## Abstract

The Age-Friendly Health System (AFHS) evidence-based 4Ms framework (What Matters, Medication, Mentation, & Mobility) is designed to be implemented in clinical settings to reliably provide high-quality age-friendly care to older adults and reduce harm and hospital-associated complications. The 4M concept of ‘What Matters to You’ (WMTY) refers to assessing and aligning an individual’s specific health outcome goals and care preferences to ensure patient-centered care with a measurable impact on patient experience. While published toolkits support the implementation of WMTY for all disciplines, there is limited guidance and empirical evidence about the implementation and impact of this concept when used by clinical nurses in acute care. Some researchers have identified issues with implementing WMTY with patients who are not able to articulate their wishes or when the question is posed in a granular way. A large healthcare system in the Midwest supported five hospitals to implement the AFHS 4Ms including the WMTY concept but did not observe an improvement in nurse-sensitive patient experience measures. A qualitative study is in progress aimed at gaining an in-depth understanding of the knowledge, perceptions, and experiences of leaders and clinical nurses who have implemented the WMTY concept for older adults in their hospital to evaluate the implementation and impact on patient care. The results gathered by this study will lay the foundation for designing clinical nurse-targeted interventions to successfully implement WMTY and identifying supportive strategies for partnering with patients and their caregivers to ensure that their goals/preferences are fully integrated into care.

